# Association Between ABO Blood Group and Survival Outcomes in Advanced NSCLC Treated with Immune Checkpoint Inhibitors

**DOI:** 10.3390/curroncol33100590

**Published:** 2026-09-30

**Authors:** Hilal Karakaş, Safa Can Efil, Burak Bilgin, Mehmet Ali Nahit Şendur, Murat Bardakçı, Perihan Perkin, Yakup Ergun

**Affiliations:** 1 Department of Medical Oncology, Ankara Bilkent City Hospital, Ankara 06800, Türkiye; safacan.efil@saglik.gov.tr (S.C.E.); burakbilgin@aybu.edu.tr (B.B.); perihan.perkin@saglik.gov.tr (P.P.); 2 Department of Medical Oncology, Diyarbakır Gazi Yaşargil Training and Research Hospital, Diyarbakır 21070, Türkiye; murat.bardakci@saglik.gov.tr; 3 Department of Medical Oncology, Bower Hospital, Diyarbakır 21070, Türkiye; dr.yakupergun@gmail.com

**Keywords:** non-small cell lung cancer, ABO blood group, immunotherapy, immune checkpoint inhibitors

## Abstract

Background: Reliable biomarkers that predict outcomes with immune checkpoint inhibitors (ICIs) in non-small cell lung cancer (NSCLC) remain limited. ABO blood group antigens may influence tumor immunity and response to immunotherapy. Methods: We retrospectively analyzed 239 patients with advanced NSCLC treated with ICI-based therapy between 2019 and 2024. Patients were classified according to ABO blood group, and overall survival (OS), progression-free survival (PFS), and treatment response were evaluated. Results: Patients with blood group O had significantly longer OS (18.6 vs. 11.0 months; *p* < 0.001) and PFS (9.4 vs. 4.2 months; *p* = 0.001) than patients with non-O blood groups. In multivariable analyses, blood group O remained independently associated with improved OS and PFS. Conclusions: Blood group O may represent a simple and readily available host-related prognostic marker in patients with advanced NSCLC receiving ICI-based therapy. Prospective multicenter studies are required to validate these findings.

## Simple Summary

Immune checkpoint inhibitors have improved outcomes in advanced non-small cell lung cancer, but readily available prognostic markers remain limited. In this retrospective real-world study of 239 patients, blood group O was associated with longer overall and progression-free survival and remained independently associated with favorable outcomes after adjustment for treatment line and other prognostic factors. These findings suggest that ABO blood group may represent a simple host-related prognostic marker in patients receiving immune checkpoint inhibitor-based therapy.

## 1. Introduction

Lung cancer remains a major cause of cancer-related mortality worldwide, despite substantial advances in molecular characterization and treatment. The identification of actionable oncogenic alterations has transformed the therapeutic landscape of non-small cell lung cancer (NSCLC), while immune checkpoint inhibitors (ICIs), either alone or in combination with chemotherapy, have become a cornerstone of treatment for patients without targetable molecular alterations [1].

Nevertheless, clinical outcomes with ICIs vary considerably among patients. Programmed death-ligand 1 (PD-L1) expression is currently the most widely used biomarker for guiding immunotherapy decisions; however, its predictive accuracy is imperfect, and durable responses may occur across different levels of PD-L1 expression. Tumor mutational burden and several emerging biomarkers—including tumor neoantigen profiles, characteristics of the tumor immune microenvironment, co-mutation patterns, epigenetic alterations, the microbiome, and transcriptomic signatures—have also been investigated, but their routine clinical applicability remains limited [1]. Consequently, there is an unmet need for simple and readily available biomarkers that may complement established tumor-related factors and help identify patients who are more likely to derive benefit from ICI-based treatment.

In this context, host-related biological characteristics have attracted increasing interest as potential determinants of antitumor immune responses. The ABO blood group system represents a genetically determined host factor that may influence immune and inflammatory processes. Emerging clinical evidence suggests that ABO blood type may be associated with outcomes following immune checkpoint blockade across different malignancies, raising the possibility that inherited blood group characteristics could contribute to interindividual variability in immunotherapy efficacy [2,3].

ABO antigens are not restricted to red blood cells but are also expressed on platelets, leukocytes, plasma proteins, and the epithelial surfaces of several organs, including the lung. Alterations or loss of ABO antigen expression have been described during malignant transformation, suggesting a potential relationship between the ABO system and tumor biology [4]. Moreover, associations between ABO blood groups and the risk or prognosis of several malignancies, including gastric, pancreatic, and hepatocellular cancers, have been reported [5]. However, compared with the extensive literature examining cancer susceptibility, evidence regarding the prognostic significance of ABO blood groups in patients receiving anticancer therapy, particularly ICIs, remains scarce.

Therefore, we aimed to investigate the association between ABO blood group and clinical outcomes in a real-world cohort of patients with advanced NSCLC treated with ICI-based therapies. Specifically, we evaluated whether ABO blood group was associated with overall survival, progression-free survival, and treatment response across different treatment modalities and PD-L1 expression levels. By examining an easily accessible and routinely available host-related characteristic, this study sought to explore the potential prognostic value of ABO blood group in the immunotherapy setting.

## 2. Materials and Methods

### 2.1. Study Design and Patient Population

This retrospective, single-center cohort study included patients with advanced non-small cell lung cancer (NSCLC) who received immune checkpoint inhibitor (ICI)-based therapy and were followed at the Department of Medical Oncology, Ankara Bilkent City Hospital, between 2019 and 2024. Patients treated with either ICI monotherapy or ICI in combination with chemotherapy were eligible, irrespective of whether immunotherapy was administered in the first- or second-line setting.

Patients aged ≥ 18 years with histologically confirmed NSCLC, available clinical follow-up data, and documented ABO blood group were included. Patients who did not meet the inclusion criteria or had a concurrent second primary malignancy were excluded.

### 2.2. Data Collection and Treatment Characteristics

Demographic, clinical, pathological, and laboratory data were retrospectively obtained from electronic medical records. The recorded variables included age, sex, smoking status, Eastern Cooperative Oncology Group (ECOG) performance status, body mass index (BMI), histological subtype, PD-L1 expression, number and sites of metastases, Charlson Comorbidity Index (CCI), ABO and Rh blood groups, neutrophil-to-lymphocyte ratio (NLR), ICI regimen, treatment modality (ICI monotherapy or ICI plus chemotherapy), and line of ICI therapy.

ECOG performance status was categorized as 0–1 versus 2–3. The ICI treatment line was categorized as first-line (1L) or second-line (2L) therapy. All eligible patients receiving ICI-based treatment in either treatment line were included in the analysis.

PD-L1 expression was categorized as negative (<1%) or positive (≥1%). Patients with unknown PD-L1 status were analyzed as a separate category. Because of the limited number of patients with PD-L1 expression ≥ 50%, this subgroup was not analyzed separately.

### 2.3. Treatment Response and Survival Outcomes

Patients underwent clinical examination and hematological and biochemical assessments before each treatment cycle. Radiological tumor assessment was performed using computed tomography (CT) or positron emission tomography/computed tomography (PET/CT), generally at 12-week intervals, and treatment response was evaluated according to the Response Evaluation Criteria in Solid Tumors (iRECIST).

Overall survival (OS) was defined as the time from the initiation of ICI-based therapy to death from any cause or the date of last follow-up. Progression-free survival (PFS) was defined as the time from the initiation of ICI-based therapy to radiological or clinical disease progression or death, whichever occurred first. Patients without an event were censored at the date of their last follow-up.

### 2.4. Statistical Analysis

Statistical analyses were performed using IBM SPSS Statistics for Windows, version 22.0 (IBM Corp., Armonk, NY, USA). Continuous and categorical variables were summarized using appropriate descriptive statistics. Survival outcomes were estimated using the Kaplan–Meier method, and differences between groups were compared using the log-rank test.

Univariable and multivariable Cox proportional hazards regression analyses were performed to identify factors associated with OS and PFS. Variables considered clinically relevant and/or associated with survival in univariable analyses were evaluated in the multivariable models. Given the potential influence of treatment line on survival outcomes, the line of ICI therapy (first-line vs. second-line) was additionally incorporated into the multivariable Cox regression models to account for its potential confounding effect on the association between ABO blood group and survival. Hazard ratios (HRs) with 95% confidence intervals (CIs) were reported.

Based on its widespread use in previous retrospective studies, an NLR cut-off value of 5 was used to categorize patients as NLR < 5 or ≥5. A two-sided *p*-value < 0.05 was considered statistically significant.

## 3. Results

### Patient Characteristics

Among the 239 patients included in the study, the median age was 64 years (range, 25–82), and 84.9% (n = 203) were male. ECOG performance status was 0–1 in 81.2% (n = 194) and 2–3 in 18.8% (n = 45) of patients.

A balanced distribution was observed when patients were evaluated according to their BMI. Baseline characteristics were generally well balanced between the groups, except for treatment line and number of treatment cycles. A substantial proportion of patients had unknown PD-L1 status due to the retrospective nature of the study.

Of the patients, 90.4% (n = 216) were active or former smokers, whereas 9.6% (n = 23) were never-smokers. Non-squamous histology was present in 62.3% (n = 149). PD-L1 expression was ≥1% in 43.5% (n = 104), <1% in 20.1% (n = 48), and unknown in 36.4% (n = 87). Most patients (92.1%, n = 220) had metastatic disease. The median number of ICI treatment cycles was 8 (range, 1–85); patients with blood group O received a median of 12 cycles, compared with 7 cycles in the non-O group. ICI monotherapy was administered to 64.9% (n = 155) of patients.

Pembrolizumab was administered to 44.8% (n = 107) of patients and nivolumab to 40.2% (n = 96); the remaining patients received nivolumab plus ipilimumab, atezolizumab, or durvalumab.

At baseline, 65.7% (n = 157) of patients had an NLR < 5, whereas 34.3% (n = 82) had an NLR ≥ 5.

Baseline patient characteristics are summarized in Table 1.

## 4. Survival Results

At a median follow-up period of 48.85 months (95% confidence interval [CI], 27.67–70.03), clinical or radiological progression or death occurred in 204 patients (85.4%). Of these patients, 185 (77.4%) had died.

The median overall survival (OS) for all patients was 12.87 months (95% CI: 10.01–15.73), and the median progression-free survival (PFS) was 6.14 months (3.95–8.33).

Patients with blood group O had significantly longer OS than patients with other blood groups (median OS: 18.56 vs. 11.03 months; *p* < 0.001) (Figure 1A). Similarly, PFS was significantly longer in the O group (median PFS: 9.42 vs. 4.17 months; *p* = 0.001) (Figure 1B). In multivariate analysis, blood group O remained an independent predictor of both improved OS and PFS. Patients with blood group O had a 39% reduction in the risk of death compared to non-O blood groups.

In the multivariable Cox regression analysis, blood group O remained independently associated with improved survival outcomes after adjustment for treatment line and other relevant prognostic factors. Blood group O was independently associated with a lower risk of death (HR = 0.61, 95% CI: 0.43–0.88; *p* = 0.008) and disease progression (HR = 0.69, 95% CI: 0.50–0.96; *p* = 0.029). Although treatment line was significantly associated with OS and PFS in univariable analyses, it did not retain statistical significance in the multivariable models. These findings indicate that the favorable association between blood group O and survival outcomes was independent of the line of ICI therapy.

Elevated NLR (≥5) was associated with significantly worse survival outcomes and remained an independent prognostic factor for both OS (HR = 1.39, *p* = 0.036) and PFS (HR = 1.38, *p* = 0.029). In contrast, PD-L1 expression was not significantly associated with overall survival (*p* = 0.467) or progression-free survival (*p* = 0.954).

A total of 239 patients were included in the survival analyses for OS and PFS. Among these, 204 patients had available radiological response assessments according to iRECIST criteria and were included in the response analyses. Accordingly, ORR and DCR were calculated based on the 204 response-evaluable patients.

Among the 204 response-evaluable patients, 2 (1.0%) achieved a complete response, 77 (37.7%) achieved a partial response, 70 (34.3%) had stable disease, and 55 (27.0%) had progressive disease. The objective response rate was significantly higher in the blood group O group than in the non-O group (53.3% vs. 33.3%; *p* = 0.008). The disease control rate was numerically higher in the blood group O group (83.3% vs. 70.2%), although the difference did not reach statistical significance (*p* = 0.052) (Table 2 and Table 3).

## 5. Discussion

In the present study, we evaluated the impact of ABO blood group on treatment response in patients with non-small cell lung cancer (NSCLC) receiving immunotherapy and demonstrated that patients with blood group O had significantly longer overall survival (OS) and progression-free survival (PFS). Importantly, the favorable association of blood group O with both OS and PFS persisted after adjustment for treatment line and other relevant prognostic factors, supporting its potential role as an independent prognostic factor in this population.

To our knowledge, this is one of the few studies evaluating the prognostic impact of ABO blood groups specifically in NSCLC patients treated with immune checkpoint inhibitors across different PD-L1 expression levels.

Numerous retrospective studies have explored the association between ABO blood groups and cancer incidence. For example, blood group A has been reported to be associated with an increased risk of gastric cancer [5]. However, studies investigating the relationship between ABO blood groups and treatment response remain limited.

In a study conducted by Chen et al., patients receiving immunotherapy were evaluated, and it was reported that patients with blood group O had an advantage in terms of time to treatment failure. However, this benefit was not reflected in survival outcomes, likely due to the heterogeneity of the study population [3]. In contrast, in our study, blood group O was consistently associated with improved survival and emerged as an independent predictor of immunotherapy response.

An important consideration in interpreting our findings is the imbalance in treatment line between the blood groups, with patients with blood group O more frequently receiving ICIs in the first-line setting. Since treatment line may influence survival outcomes, this difference could potentially confound the observed association between ABO blood group and prognosis. However, after incorporating treatment line into the multivariable Cox regression models, blood group O remained independently associated with improved OS and PFS. These findings suggest that the survival advantage observed in patients with blood group O was not solely attributable to differences in treatment line and further support the potential independent prognostic value of ABO blood groups.

Recent studies focusing on immunotherapy-treated lung cancer populations have further supported the prognostic role of ABO blood groups. In one such study, consistent with our findings, blood group O was found to be significantly associated with improved OS and PFS in patients with metastatic NSCLC who had PD-L1 expression ≥50% and were treated with immunotherapy monotherapy [2]. However, in that study, all patients were in the metastatic setting with high PD-L1 expression, and this survival advantage was not observed in patients receiving chemoimmunotherapy. In contrast, in our cohort, independent of PD-L1 expression, blood group O was shown to be a potential independent predictor of immunotherapy response in patients with stage III–IV lung cancer.

In addition to the survival advantage, patients with blood group O had a significantly higher ORR than those with non-O blood groups. However, response analyses were restricted to the 204 response-evaluable patients, and the difference in DCR did not reach statistical significance. Therefore, these response findings should be interpreted cautiously and require confirmation in prospective cohorts.

The biological mechanisms underlying the relationship between ABO blood groups and cancer outcomes have not yet been fully elucidated. ABO antigens are expressed on epithelial and endothelial cells and may influence tumor–immune interactions, inflammation, and immune cell trafficking. Differences in host inflammatory status related to ABO blood group antigens may partially explain the survival advantage observed in patients with blood group O in our cohort [6]. Polymorphisms at the ABO gene locus have been associated with circulating levels of inflammatory mediators, including tumor necrosis factor-alpha, soluble intercellular adhesion molecule-1 (ICAM-1), and selectins, all of which play a role in tumor progression and metastasis [7]. Higher ICAM-1 expression observed in non-O blood groups may further support the favorable prognostic impact of blood group O.

In addition to blood group, elevated neutrophil-to-lymphocyte ratio (NLR) was identified as an independent prognostic factor in our study, whereas PD-L1 expression was not associated with survival outcomes. Elevated NLR reflects a pro-inflammatory systemic environment and has been associated with impaired anti-tumor immune response, which may explain its negative impact on survival. In contrast, although PD-L1 expression is widely used as a predictive biomarker in immunotherapy, it was not associated with survival outcomes in our cohort. This finding may be related to the retrospective design, heterogeneity in treatment lines, and the relatively high proportion of patients with unknown PD-L1 status, which may have masked its prognostic significance.

Our findings are consistent with previous reports suggesting that blood group O may confer a survival advantage in patients receiving immunotherapy [3]. However, conflicting results have been observed in other tumor types. For instance, Ergun et al. reported improved survival outcomes in patients with blood group B in malignant melanoma, while studies in renal cell carcinoma did not demonstrate a significant association between blood group and treatment response [8]. These discrepancies may be explained by tumor-specific biological differences and variations in study populations.

Our study has several strengths, including a relatively large patient cohort, an extended follow-up period, and the use of multivariate analyses to adjust for potential confounders, thereby strengthening the validity of our findings. However, several limitations should be acknowledged. First, the retrospective and single-center design may limit the generalizability of the results. Second, the high proportion of patients with unknown PD-L1 status may have influenced the analysis. Third, we were unable to evaluate the underlying biological mechanisms due to the lack of molecular and laboratory data.

To our knowledge, this is the first study evaluating the prognostic impact of ABO blood group across all immunotherapy-based treatment modalities and PD-L1 expression levels in a real-world NSCLC population.

Additionally, heterogeneity in ICI treatment lines and treatment modalities represents a potential source of confounding, although treatment line was incorporated into the multivariable survival models. Furthermore, treatment response could be evaluated in 204 of the 239 patients, which may have introduced selection bias into the response analyses.

## 6. Conclusions

Blood group O was independently associated with improved overall and progression-free survival in patients with advanced NSCLC receiving immune checkpoint inhibitor-based therapy. These findings suggest that ABO blood group may represent a simple and readily available host-related prognostic marker; however, prospective multicenter validation is warranted.

## Figures and Tables

**Figure 1:**
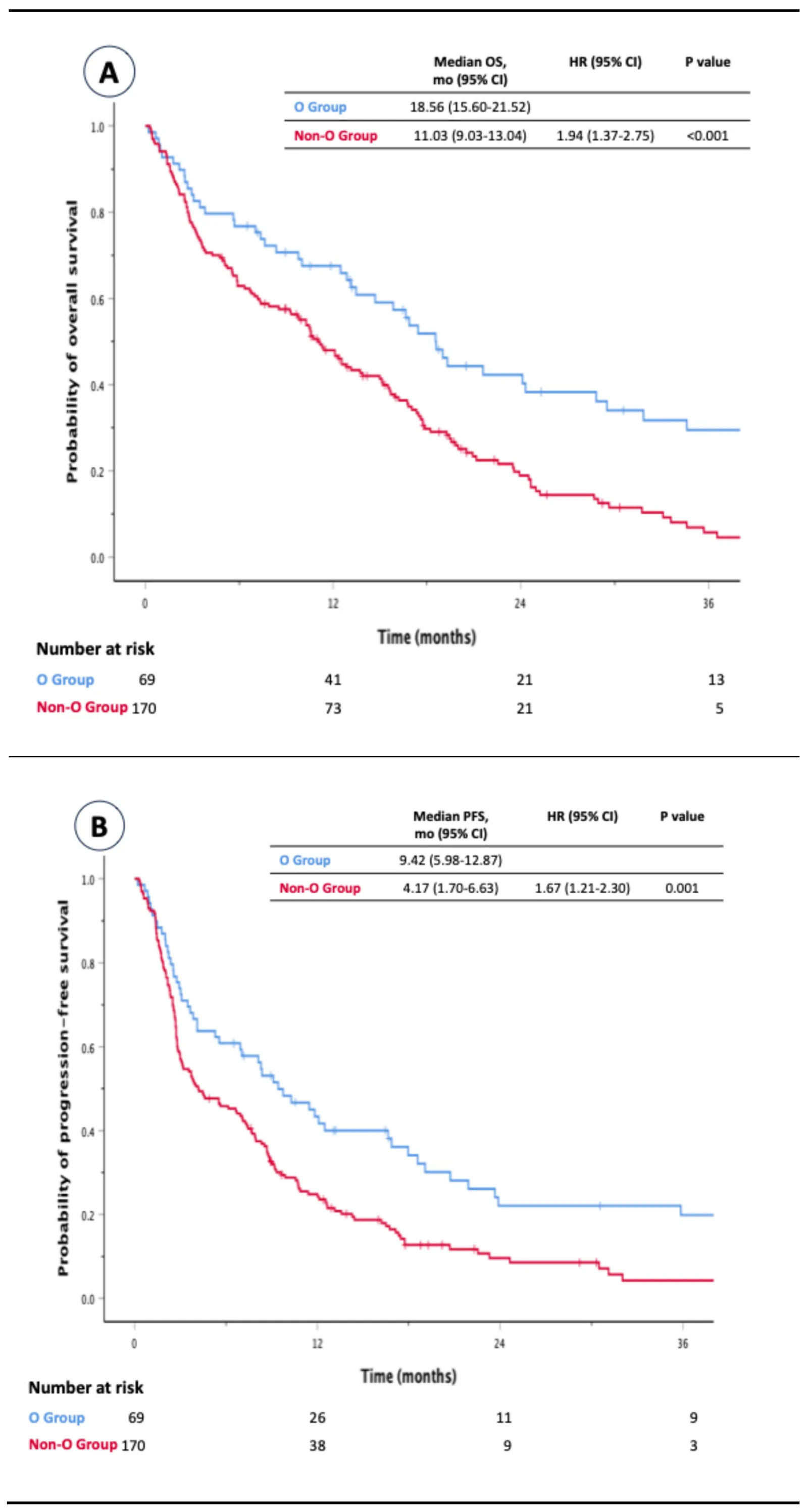
Kaplan–Meier survival curves according to ABO blood group: (**A**) overall survival and (**B**) progression-free survival in patients with advanced NSCLC receiving immune checkpoint inhibitor-based therapy.

**Table 1:** Baseline demographic, clinical, and treatment characteristics according to ABO blood group.

Characteristics		All Patients n = 239 (100%)	Type O n = 69 (29%)	Type Non-O n = 170 (71%)	*p* *
Age, median, min-max		64 (25–82)	64 (25–80)	63 (30–82)	0.551
Age, n (%)	<65 years	142 (59.4)	37 (53.6)	105 (61.8)	0.245
	≥65 years	97 (40.6)	32 (46.4)	65 (38.2)	
Gender, n (%)	Female	36 (15.1)	13 (18.8)	23 (13.5)	0.298
	Male	203 (84.9)	56 (81.2)	147 (86.5)	
ECOG PS, n (%)	0–1	194 (81.2)	59 (85.5)	135 (79.4)	0.275
	2–3	45 (18.8)	10 (14.5)	35 (20.6)	
BMI (kg/m^2^), n (%)	<25	118 (50.4)	34 (50.7)	84 (50.3)	0.951
	≥25	116 (49.6)	33 (49.3)	83 (49.7)	
Smoking, n (%)	No	23 (9.6)	9 (13.0)	14 (8.2)	0.253
	Yes	216 (90.4)	60 (87.0)	156 (91.8)	
CCI score, median, min-max		8 (3–14)	8 (3–14)	8 (3–13)	0.689
CCI score, n (%)	≤Median	126 (52.7)	35 (50.7)	91 (53.5)	0.694
	>Median	113 (47.3)	34 (49.3)	79 (46.5)	
Histology, n (%)	Non-squamous	149 (62.3)	42 (60.9)	107 (62.9)	0.765
	Squamous	90 (37.7)	27 (39.1)	63 (37.1)	
PD-L1 expression, n (%)	<1%	48 (20.1)	14 (20.3)	34 (20.0)	0.999
	≥1%	104 (43.5)	30 (43.5)	74 (43.5)	
	Unknown	87 (36.4)	25 (36.2)	62 (36.5)	
Stage, n (%)	3B-3C	19 (7.9)	6 (8.7)	13 (7.6)	0.786
	Metastatic	220 (92.1)	63 (91.3)	157 (92.4)	
No. of metastatic sites, n (%)	0–1	108 (45.2)	37 (53.6)	71 (41.8)	0.095
	≥2	131 (54.8)	32 (46.4)	99 (58.2)	
Line of ICI therapy, n (%)	1L	118 (49.4)	42 (60.9)	76 (44.7)	**0.024**
	2L	121 (50.6)	27 (39.1)	94 (55.3)	
No. of ICI treatment cycles, median, min-max		8 (1–85)	12 (1–85)	7 (1–60)	0.008
Treatment modality, n (%)	ICI-based therapy without CT	155 (64.9)	40 (58.0)	115 (67.6)	0.156
	ICI-based therapy with CT	84 (35.1)	29 (42.0)	55 (32.4)	
ICI regimen, n (%)	Pembrolizumab Nivolumab Nivolumab + Ipilimumab Atezolizumab Durvalumab	107 (44.8) 96 (40.2) 3 (1.3) 28 (11.7) 5 (2.1)	38 (55.1) 21 (30.4) 1 (1.4) 8 (11.6) 1 (1.4)	69 (40.6) 75 (44.1) 2 (1.2) 20 (11.8) 4 (2.4)	0.307
NLR	<5	157 (65.7)	51 (73.9)	106 (62.4)	0.088
	≥5	82 (34.3)	18 (26.1)	64 (37.6)	

* *p* values were calculated using the chi-square or Mann–Whitney U test, as appropriate.

**Table 2:** Objective response rate and disease control rate according to ABO blood group among response-evaluable patients.

	Type O	Type Non-O	*p*
Objective response rate (%)	53.3	33.3	0.008
Disease control rate (%)	83.3	70.2	0.052

**Table 3:** Univariable and multivariable Cox regression analyses for overall survival and progression-free survival.

OS				
Variables	Univariate		Multivariate	
	HR (95% CI)	*p* Value	HR (95% CI)	*p* Value
Age (<65 y vs. ≥65 y)	1.16 (0.86–1.56)	0.313		
Gender (Female vs. Male)	0.94 (0.63–1.41)	0.780		
ECOG (0–1 vs. 2–3)	4.72 (3.28–6.79)	<0.001	3.99 (2.71–5.88)	<0.001
BMI (<25 vs. ≥25)	1.15 (0.86–1.55)	0.332		
Smoking (Yes vs. No)	1.13 (0.70–1.82)	0.606		
CCI score (≤Median vs. >Median)	1.23 (0.92–1.64)	0.159	1.12 (0.83–1.50)	0.448
Histology (Non-SCC vs. SCC)	0.96 (0.71–1.29)	0.800		
PD-L1 expression (<1% vs. ≥1%)	0.86 (0.58–1.27)	0.467		
No. of metastatic sites (1 vs. ≥2)	2.01 (1.48–2.72)	<0.001	1.51 (1.09–2.09)	0.012
Blood group (Non-O vs. O)	0.51 (0.36–0.72)	<0.001	0.61 (0.43–0.88)	0.008
NLR (<5 vs. ≥5)	1.39 (1.03–1.88)	0.029	1.39 (1.02–1.91)	0.036
PFS				
Variables	Univariate		Multivariate	
	HR (95% CI)	*p* value	HR (95% CI)	*p* value
Age (<65 y vs. ≥65 y)	1.12 (0.85–1.48)	0.412		
Gender (Female vs. Male)	0.87 (0.59–1.29)	0.512		
ECOG (0–1 vs. 2–3)	4.55 (3.17–6.52)	<0.001	3.83 (2.65–5.53)	<0.001
BMI (<25 vs. ≥25)	1.09 (0.83–1.44)	0.516		
Smoking (Yes vs. No)	1.21 (0.77–1.91)	0.392		
CCI score (≤Median vs. >Median)	1.17 (0.88–1.54)	0.267	0.98 (0.74–1.30)	0.907
Histology (Non-SCC vs. SCC)	0.96 (0.72–1.28)	0.814		
PD-L1 expression (<1% vs. ≥1%)	0.98 (0.68–1.43)	0.954		
No. of metastatic sites (1 vs. ≥2)	2.20 (1.63–2.96)	<0.001	1.79 (1.31–2.45)	<0.001
Blood group (Non-O vs. O)	0.59 (0.43–0.82)	0.002	0.69 (0.50–0.96)	0.029
NLR (<5 vs. ≥5)	1.57 (1.18–2.09)	0.002	1.38 (1.03–1.85)	0.029
ABO blood group	0.534	<0.001	0.632	0.005
Line of ICI therapy	0.771	0.082	0.617	<0.001

## Data Availability

The data supporting the findings of this study are available from the corresponding author upon reasonable request, subject to institutional and ethical restrictions.

## References

[1] Meyer M.-L., Fitzgerald B.G., Paz-Ares L., Cappuzzo F., A Jänne P., Peters S., Hirsch F.R. (2024). New promises and challenges in the treatment of advanced non-small-cell lung cancer. Lancet.

[2] Certa F., Horn P.A., Keyl J., Mende B., Lueong S., Hilser T., Theurer S., Virchow I., Zaun Y., Pogorzelski M. (2025). ABO-Blood Group Associates with Survival Outcomes in Patients with Metastatic Non-Small Cell Lung Cancer Treated with Pembrolizumab Monotherapy. Thorac. Cancer.

[3] Chen R., Hakimi K., Zhang X., Messer K., Patel S.P. (2022). Patient Blood Type Is Associated with Response to Immune Checkpoint Blockade in Metastatic Cancer. Oncologist.

[4] Luo Q., Pan M., Feng H., Wang L. (2021). ABO blood group antigen therapy: A potential new strategy against solid tumors. Sci. Rep..

[5] Wang Z., Liu L., Ji J., Zhang J., Yan M., Zhang J., Liu B., Zhu Z., Yu Y. (2012). Abo blood group system and gastric cancer: A case-control study and meta-analysis. Int. J. Mol. Sci..

[6] Engin H., Bilir C., Üstün H., Gökmen A. (2012). ABO blood group and risk of pancreatic cancer in a Turkish population in Western Blacksea region. Asian Pac. J. Cancer Prev..

[7] Franchini M., Liumbruno G.M., Lippi G. (2015). The prognostic value of ABO blood group in cancer patients. Blood Transfus..

[8] Ergun Y., Esen S.A., Bardakci M., Ucar G., Kalkan Z., Urakci Z., Seyran E., Dogan M., Eren T., Aslan V. (2022). Predictive and prognostic effect of ABO blood group on immune checkpoint inhibitors. Cancer Biomark..

